# Development of a culturally enhanced caregiver-facilitated language nutrition intervention “+Language is Medicine” to address developmental delay in Diné (Navajo) toddlers

**DOI:** 10.3389/fpubh.2024.1376742

**Published:** 2024-06-19

**Authors:** Taylor Billey, Elizabeth Kushman, Jessica Meese, Lisa Martin, Lisa Jim, Martha A. Austin-Garrison, Joshuaa D. Allison-Burbank

**Affiliations:** ^1^Center for Indigenous Health, Department of International Health, Johns Hopkins Bloomberg School of Public Health, Baltimore, MD, United States; ^2^Center for Diné Studies, Diné College, Shiprock, NM, United States

**Keywords:** American Indian/Alaska native, indigenous, early childhood, developmental delay, infants, toddlers, tribal home visiting, indigenous language nutrition

## Abstract

**Introduction:**

Developmental Delay (DD) is highly common in American Indian and Alaska Native (AI/AN; Indigenous) toddlers and leads to high numbers of AI/AN children who eventually need special education services. AI/AN children are 2.89 times more likely to receive special education compared to other children in the U.S., yet developmental disorders are more frequently under diagnosed and untreated in AI/AN infants and toddlers. DD, which can be identified as early as toddlerhood, can lead to negative impacts on developmental trajectories, school readiness, and long-term health. Signs of DD can be identified early with proper developmental screening and remediated with high quality early intervention that includes effective parent training. There are many evidence-based language facilitation interventions often used in Early Intervention programs. However, in communities in rural parts of the Navajo Nation where there are limited services and resources, infants and toddlers with early signs of DD are often missed and do not get the culturally responsive support and evidence-based intervention they deserve.

**Methods:**

The community-based +Language is Medicine (+LiM) study team partnered with tribal home visitors, community members, and a Diné linguist/elder using a collaborative virtual workgroup approach in 2021 and 2022 to present the +LiM pilot study aims and to discuss strategies for enhancing a language intervention for toddlers experiencing DD in their tribal community. This paper will detail the stages of community engagement, intervention enhancement and preparation for field testing of the +LiM intervention to address elevated rates of DD in toddlers in the Northern Agency of the Navajo Nation.

**Results:**

Two major outcomes from this collaborative workgroup included: (1) a team-initiated redefining of language nutrition to align with Indigenous values that center cultural connectedness and native language use and (2) a five-lesson caregiver-facilitated curriculum titled +Language is Medicine which includes caregiver lessons on language nutrition, language facilitation, shared book reading, pretend play, and incorporation of native language into home routines. These two workgroup outcomes were leveraged to develop a pilot pre−/post-intervention study to test the effectiveness of the +LiM intervention with caregiver-toddler dyads living on the Navajo Nation.

**Discussion:**

Delivering tailored child interventions through tribal home visiting are cost-effective and innovative methods for reaching reservation-based families who benefit from culturally responsive parent coaching and instruction. The +LiM team has applied a precision tribal home visiting approach to enhance methods of early intervention for children with DD. Our enhancement process was grounded in Indigenous community-based participatory research that centered culture and language.

## 1 Introduction

“You must speak straight so that your words may go as sunlight into our hearts.”

~ Cochise, Chiricahua

American Indian and Alaska Native (AI/AN; Indigenous) people make up highly language-rich communities that are full of stories, ceremonies, celebration, and humor. These communities continue to thrive through their oral traditions despite a long history of trauma and genocide brought on by outsiders who colonized their traditional homelands. Today, the effects of these traumas continue to impact parental responsiveness and attachment style which negatively impacts childrearing practices and communications styles with young children (1). It is through this context that we must explore child well-being and work toward trauma-informed and culturally responsive early childhood interventions that can steer early developmental trajectory in positive directions. Language-rich experiences are common in AI/AN households with strong oral traditions, Furthermore, it is established that these Indigenous families who maintain strong connections to tradition often engage in cultural routines that bring together family and community in which there is frequent language exchange. Therefore, this study team sought a family and community-level intervention approach that built on increasing positive parenting, attachment with young children, and increase of language nutrition. Language nutrition is a concept used to describe early language exposure that is rich in quality and quantity and aligns with a family’s cultural and home routines (2). It is through the concept that this study and the cultural enhancements of early language facilitation strategies were planned to meet the rising incidence of developmental delay (DD) on the Navajo Nation.

### 1.1 Background

**Figure 1:**
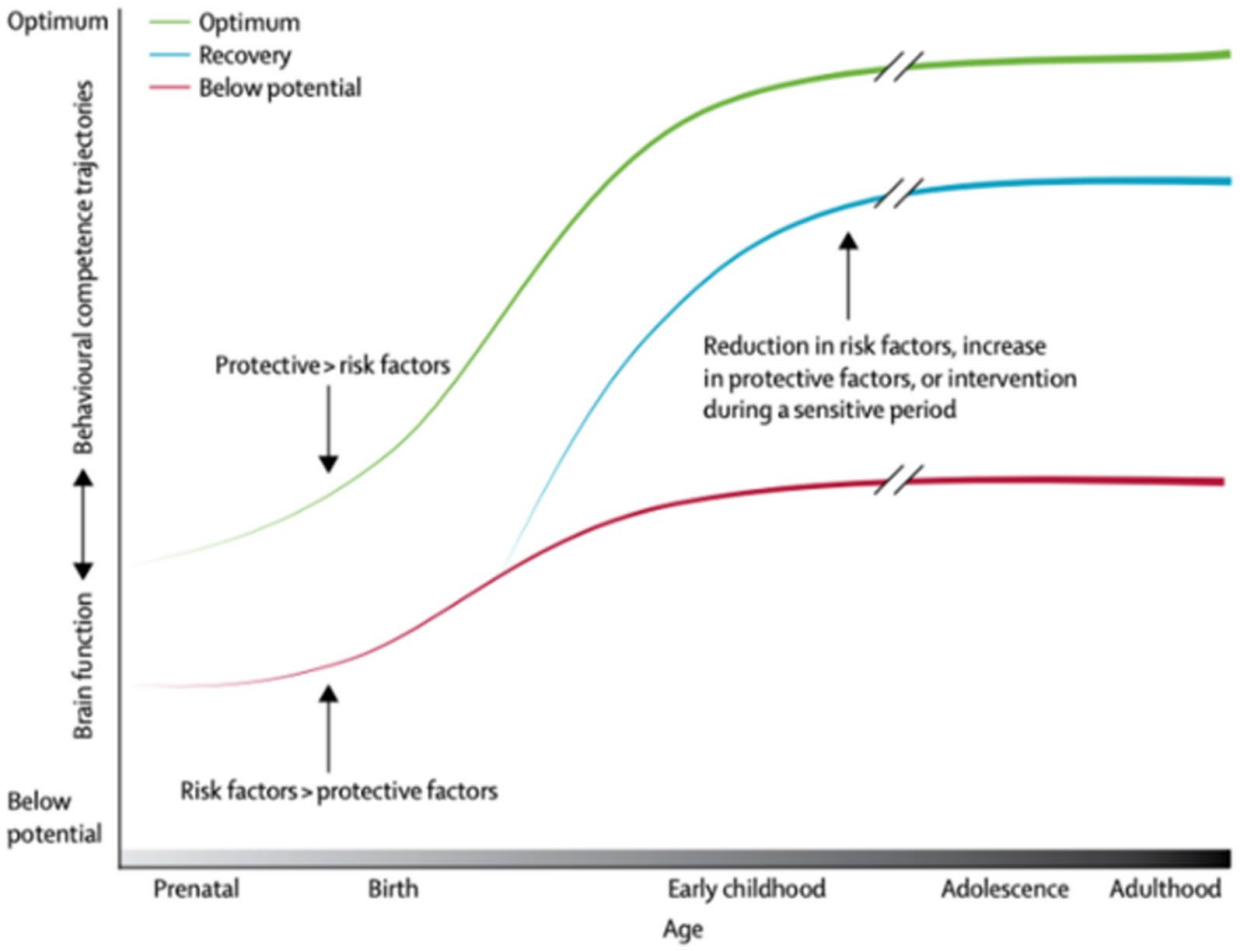
Brain function and behavioral competency trajectories across the lifespan.

DD, which is defined as significant delays in two or more developmental domains of communication, problem solving, social emotional, motor, and self-help (3), has become highly common in AI/AN infants and toddlers. This has led to an overrepresentation in Individuals with Disabilities Education Act (IDEA) Part B and C programs with the higher incidence staying high following the COVID-19 pandemic. AI/AN children are 2.89 times more likely to receive special education services compared to other children in the United States (U.S.), yet neurodevelopmental disorders, including developmental delay, are more frequently underdiagnosed and untreated in AI/AN children (4, 5), DD, which can be identified as early as infancy and toddlerhood, can lead to negative impacts on developmental trajectories, school readiness, and long-term health (6, 7). Signs of DD can be identified early with proper screening and remediated with high quality early intervention that includes parent training (8). However, unequitable policies in states with many AI/AN children result in many referred children missing the opportunity for early intervention services. For example, in Arizona, children must have an established medical condition or a delay of at least two standard deviations (approximately 50 percent below the mean) in at least one developmental area, resulting in 66% of referred children not qualifying for services (9). For children who do qualify, their families often face long waiting times due to a lack of providers and system barriers that lead to delayed intervention and culturally responsive parental supports. Risk factors such as low socioeconomic status, low parental responsivity, and language-poor environments can limit early brain functioning and language development (10). However, protective factors include high parental responsivity, high print exposure, longer reciprocal caregiver-child interactions, and increased exposure to rich language and foster timely achievement of developmental milestones (11). Figure 1 (12), depicts how risk factors reduce long-term developmental trajectory and highlights the optimal timing for intervention to reduce risk factors for DD is shortly after birth to age 5 (13).

Addressing DD in early childhood can help prevent health inequities that manifest throughout adulthood. Risk factors for DD include decreased structured play with caregivers and reduced parent responsiveness. Children with DD encounter multiple academic and social challenges elevating their risk for chronic and behavioral health conditions later in life. The link between early developmental trajectories and health inequities is well established (14–16) to address this significant disparity, earlier identification of DD and expanded opportunities for early remediation are needed.

The Navajo Nation, the largest reservation in the United States, is a vast and culturally rich territory spanning portions of Arizona, New Mexico, and Utah. Home to the *Diné* people, the nation boasts a population that surpasses 300,000, making it one of the most populous Indigenous communities. Known for their strong emphasis on traditional values of lifelong learning and reflection, the Diné people have a deep connection to the land, with a rich cultural heritage reflected in their art, language, and ceremonies. The Navajo Nation is renowned for its unique landscape, cornfields, valleys, and canyons. From early creation through present day, the Diné have always demonstrated resilience through initiatives focused on education, healthcare, language revitalization, and community-leveraged public health solutions. The community’s strengths lie in its commitment to preserving its language, fostering cultural continuity, and implementing sustainable practices to improve the well-being of all Diné, especially in young children (17). The demographics of the Navajo Nation showcase a diverse population, with a blend of traditional and contemporary elements contributing to the unique fabric of this large sovereign nation. A core Diné philosophy of Hózhó can help to conceptualize Diné wellness and beliefs of living intentionally and in harmony with one’s surroundings. This traditional teaching is used as a guide for how to organize the thoughts, actions, behaviors, and speech of the Diné people (18–21). When thinking about potential public health initiatives to improve child and family well-being, these characteristics, strengths, and ways of life must be considered when attempting to deliver evidence-based interventions (EBIs) in Diné households. Therefore, the overarching goal, when attempting to address early indicators of DD should be to provide evidence-based services that are culturally tailored and meet the whole child and their families (22). To accomplish this, there must be existing programmatic and workforce infrastructure in place in which the enhanced intervention can be delivered. Family support programs delivered by non-clinical, community-based providers, including Tribal Home Visiting (THV) programs, hold great promise for reaching families in underserved AI/AN communities in an effective way.

#### 1.1.1 The power of tribal home visiting

Home Visiting has been recognized as a highly effective approach for promoting cognitive and social–emotional development in young children, with multiple program models showing positive outcomes with significant effect sizes, across diverse populations. THV programs specifically have shown that training community members (who are familiar with cultural beliefs, historical trauma, and language preferences of an Indigenous community) as home visitors, is a highly effective strategy for delivering health and developmental interventions (10, 11, 13, 23, 24). Further, THV programs, which are much more accessible than specialty early intervention clinical services, address social determinants of health in AI/AN communities, and are generally well-received – suggesting they could be an effective platform for dissemination of culturally-tailored parent coaching interventions, such as +LiM, to address early signs of DD. Family Spirit is a THV program developed by the Johns Hopkins Center for Indigenous Health (JHCIH) in collaboration with the Navajo, White Mountain Apache, and San Carlos Apache tribal communities. Family Spirit is currently the only THV model categorized as evidence-based for AI/AN communities and is delivered by community-based Health Educators to support caregivers during pregnancy and early childhood. +LiM is structured to mirror the core elements of the Family Spirit home visitation model which include relationship building, defined home visitation methods, utilization of strengths-based approaches, and defined structures for delivering the material. When considering the variety of systems and services that address early childhood needs, THV emerges as a valuable but largely untapped secondary prevention approach to mitigate early developmental risks within AI/AN children, harnessing the strength of culturally sensitive, family-centered interventions. By deploying knowledgeable professionals who understand the unique cultural contexts and challenges faced by AI/AN families, THV programs can effectively identify and address early developmental risks. Home visiting initiatives focus on empowering caregivers with the skills and resources needed to foster positive early childhood experiences. The home-based nature of these interventions ensures that they are tailored to the specific needs of each family, promoting a holistic approach that integrates cultural values and traditions. Through fostering strong caregiver-child relationships and offering support in the crucial early years, THV is not only poised to address developmental risks, but also represents a restoration of traditional community member roles and responsibilities related to caring for the next generation. By passing along skills, culture, and traditions to children that experience them with their caregivers, the intervention supports learning and healing so that children acquire and share these skills and behaviors with the children that they may care for when they become adults. In this way, enhancing THV programs guided by an implementation science framework, by equipping home visitors with early intervention training and resources can contribute to the preservation and revitalization of Indigenous cultures, reinforcing the resilience and well-being of AI/AN communities while also using proven research methods that lead to long-term sustainability of enhanced evidence-based therapies.

## 2 Methods

**Figure 2:**
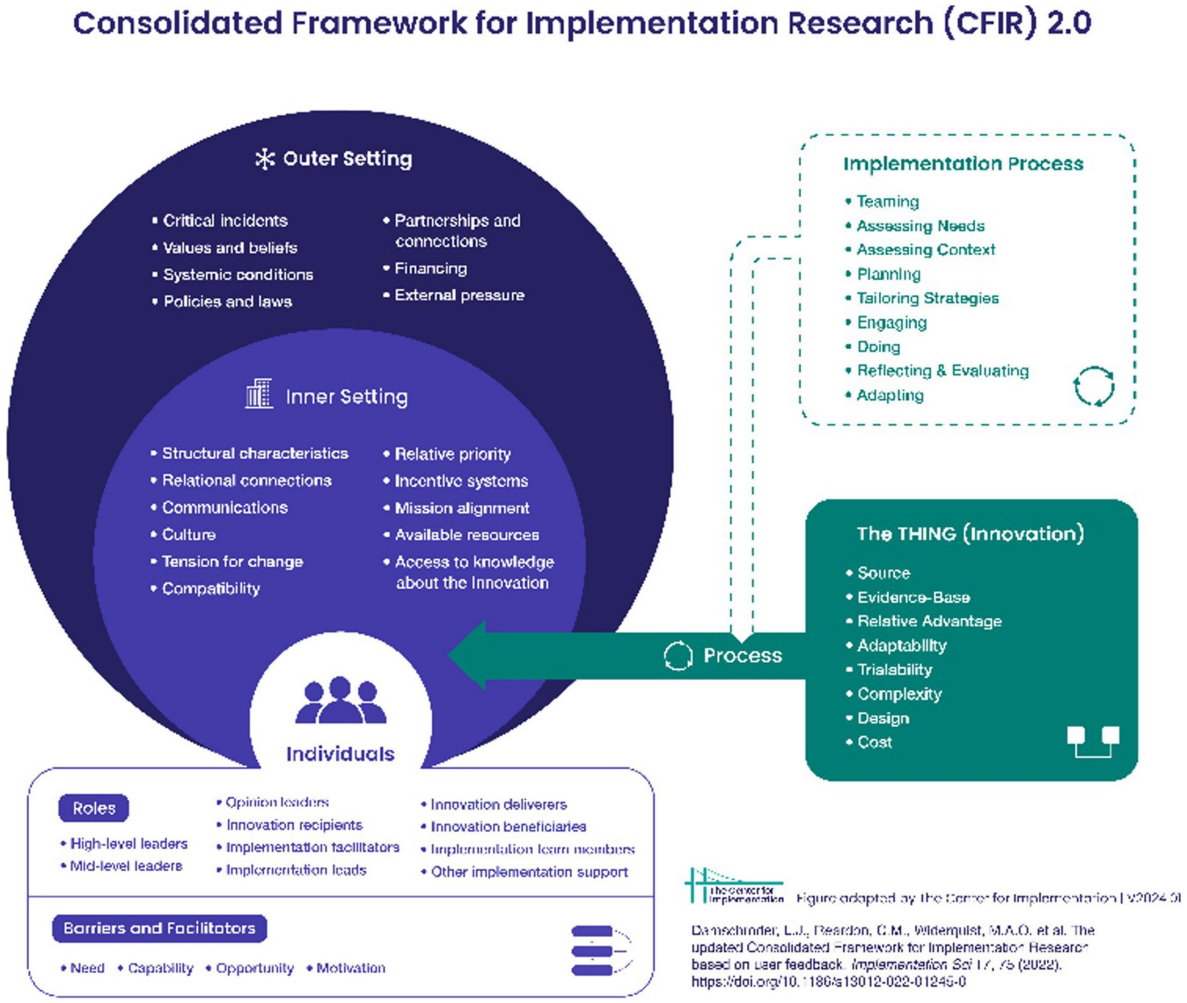
CFIR promotes consistent use of constructs, systematic analysis, and organization of findings from implementation studies.

Due to the overburdening of IDEA Part B and C services on the Navajo Nation, a focus has been on identifying novel solutions to remediate DD early in childhood rather than to allow DD to progress and complicate growth and development through childhood. This section presents the initial enhancement process for two commonly used language facilitation approaches typically delivered by speech-language pathologists (SLP) including those working in IDEA Part C early intervention programs. This work on the Navajo Nation utilizes the Consolidated Framework for Implementation Research (CFIR) to operationalize the process of study concept development through the piloting phase of a culturally enhanced intervention described below (25). See Figure 2 for CFIR framework. The use of implementation strategies helps researchers to identify potentially successful determinants of new intervention development and to strategically plan for precision delivery to diverse communities such as the Navajo Nation. Therefore, the use of the CFIR stage of The THING, which included the selected evidence-based therapies common in early intervention (Innovation) was used to guide the adaptation process, hereinafter referred to as the enhancement process, of two evidence-based therapeutic models embedded within a caregiver-facilitated intervention being piloted on the Navajo Nation. In this initial implementation stage, evidence-based therapies are identified as suitable for the population of interest (toddlers with elevated DD risk) and the process of enhancement is established with community and regional early intervention expertise. This process of enhancement, which employs a strategic process, is the focus of this paper along with consideration of decolonization which can help Indigenous researchers to deconstruct historical and contemporary elements of colonialism (26).

### 2.1 Enhancement process

The research team utilized steps identified in a scoping study, see Table 1 (27) below, of adaptation frameworks for adapting public health evidence-based interventions (EBI) to organize how the +Language is Medicine (+LiM) intervention was developed and how language facilitation strategies, the Hanen “It Takes Two to Talk” and Enhanced Milieu Teaching (EMT), were enhanced to be delivered to toddlers in the Northern Agency of the Navajo Nation. The stages discussed in this paper are as follows: (1) assess the community, (2) understand the intervention(s), (3) select the intervention(s), (4) consult with experts, (5) consult with stakeholders, (6) decide what needs adaptation, (7) enhance the original program, (8) train staff, and (9) test the enhanced materials. The +LiM intervention is currently engaged in a pilot study and therefore, the remaining steps have been omitted for detailed evaluation after pilot study completion.

**Table 1:** Key adaptation steps and descriptions.

Step name	Step descriptions
1. Assess community	Identify behavioral determinants and risk behaviors of the new target population using focus group, interviews, needs assessment, and logic models.
	Assess organizational capacity to implement the program
2. Understand the intervention	Identify and review relevant EBPs and their program materials
	Understand the theory behind the programs and their core elements
3. Select intervention	Select the program that best matches the new population and context
4. Consult with experts	Consult content experts, including original program developers, as needed
	Incorporate expert advice into program
5. Consult with stakeholders	Seek input from advisory boards and community planning groups where program implementation takes place
	Identify stakeholder partners who can champion program adoption in new setting and ensure program fidelity
6. Decide what needs adaptation	Decide whether to adapt implement original program
	Theater test selected EBP using new target population and other stakeholders to generate adaptations
	Determine how original and new target population/setting differ in terms of risk and protective factors
	Identify areas where EBP needs to be adapted and include possible changes in program structure, content, provider, or delivery methods
	Retain fidelity to core elements
	Systematically reduce mismatches between the program and the new context
7. Adapt the original program	Develop adaptation plan
	Adapt the original program contents through collaborative efforts
	Make cultural adaptations continuously through pilot testing
	Core components responsible for change should not be modified
8. Train staff	Select and train staff to ensure quality implementation
9. Test the adapted materials	Pretest adapted materials with stakeholder groups
	Conduct readability tests
	Pilot test adapted EBP in new target population
	Modify EBP further if necessary
10. Implement	Develop implementation plan based on results generated in previous steps
	Identify implementers, behaviors, and outcomes
	Develop scope, sequence, and instructions
	Execute adapted EBP
11. Evaluate	Document the adaptation process and evaluate the process and outcomes of the adapted intervention as implemented
	Write evaluation questions; choose indicators, measures, and the evaluation design; plan data collection, analysis, and reporting
	Employ empowerment evaluation approach framework to improve program implementation

#### 2.1.1 Assess the community needs

The Johns Hopkins Center for Indigenous Health (JHCIH) has continuously operated service-research sites on the Navajo Nation since the mid-1980s. Since 2013, JHCIH has operated a range of behavioral and mental health programs focusing on maternal and child health, promoting physical activity, and suicide prevention that have greatly improved the health and well-being of Diné families. JHCIH has delivered Family Spirit curriculum and developed and tested Family Spirit supplemental modules that target various health outcomes with children and their caregivers over the last 10 years in the Northern Agency of the Navajo Nation (24, 28, 29). The Northern Agency is located in the northern part of the Navajo Nation with 20 chapter houses or local governments to serve each community. Through JHCIH’s established Family Spirit presence within this region and experience utilizing Diné community members as home visitors, the research team chose Shiprock, NM as the center location for the development and field testing of the +Language is Medicine intervention. Further, community partnerships in the Northern Agency, established through previous JHCIH Family Spirit implementation efforts, have indicated an increase referral rate of children to early intervention services. Additionally, the lead PI, a licensed Diné SLP has worked with the Indian Health Service and worked with the local Part C early intervention program. It was based on these firsthand experiences that the study team shifted their attention to secondary prevention efforts to respond to the high incidence of DD in this region of the Navajo Nation.

#### 2.1.2 Understanding and selecting the EBIs

The Hanen “It Takes Two to Talk” approach (30), is an early language facilitation program designed to support caregivers in promoting language development of their young children (birth to 5 years old), particularly those with language delays or difficulties. Developed by The Hanen Centre, this evidence-based intervention focuses on empowering caregivers as the primary facilitators of their child’s communication skills. “It Takes Two to Talk” employs interactive strategies to enhance language-rich interactions within everyday routines, emphasizing the importance of responsive and child-centered communication. The program provides a step-by-step guide to educated caregivers as the primary facilitators through personalized coaching. Through this program, caregivers learn to recognize and respond effectively to their child’s cues to create an enriched language-learning environment. By incorporating practical techniques into daily interactions, the program not only promotes language development but also strengthens the careiver-child bond. This intervention is recognized for its family-centered approach which acknowledges the crucial role caregivers play in supporting their children’s communication skills during the foundational years of early childhood development.

Enhanced Milieu Teaching [EMT; (31)], is an evidence-based intervention that has proven effective in supporting toddlers with developmental delays in their language acquisition. EMT emphasizes creating an early language-rich environment within the child’s natural context. This approach involves shared book reading and structuring play interactions based on the child’s interest to encourage communication and language using naturalistic teaching strategies. With a focus on increasing the child’s initiation of communication, EMT employs prompts, expansions, and modeling techniques to enhance language development. This method not only fosters the child’s expressive and receptive language skills but also promotes generalization of these skills across various settings. EMT recognizes the importance of individualized, child-centered interventions and encourages active participation from caregivers and caregivers. EMT is a valuable tool for early intervention programs focused on supporting toddlers with developmental delays in achieving meaningful language milestones.

Incorporating shared book reading and pretend play instruction into home visiting programs is essential for fostering holistic child development and strengthening the parent/caregiver-child relationship within common caregiver-child routines. Shared book reading not only exposes children to language-rich environments but also promotes early literacy skills, vocabulary expansion, and cognitive development (32, 33). Playtime is an important opportunity for learning and brain development. Pretend play is when a child uses their imagination to do common everyday activities in their play by using an object, idea, or action to represent another object, idea, or action. (34, 35) Additionally, engaging in pretend play enhances a child’s imaginative and social–emotional skills, allowing them to explore creativity, problem-solving, and interpersonal skills. Integrating these activities into home visiting programs not only supports children’s cognitive and language development but also empowers caregivers with valuable tools to actively participate in their child’s learning journey. Both shared book reading, and pretend play create opportunities for meaningful interactions, reinforcing positive relationships between caregivers and children. By emphasizing these activities as important routines in which caregiver coaching can be caregivers, home visiting programs can contribute significantly to the overall well-being and school readiness of young children. Equipping caregivers with practical strategies through coaching and modeling promotes families to continue to support their child’s growth outside of structured intervention sessions, in a naturalistic environment.

#### 2.1.3 Select the interventions

The previously mentioned EBIs, “It Takes Two to Talk” (30), and EMT (31), were chosen by the lead PI, a licensed Diné SLP. These two language facilitation approaches are commonly used in developmental early intervention (EI) programs that include home visits and parent coaching. The parent coaching model has been mostly adopted into Birth-to-Three EI programs because of its ability to have a positive impact on parent behavior rather than direct intervention with a child (36). Therefore, these two interventions were selected to be culturally enhanced for Diné families and adapted to be delivered by supervised tribal home visitors.

#### 2.1.4 Consult with experts

Community advisory boards (CAB) play a crucial role in research with tribal communities by providing a culturally grounded and community-specific perspective (37–42). These boards help ensure that research respects tribal sovereignty, addresses community priorities, and incorporates local knowledge, fostering trust, collaboration, and the ethical conduct of research within the cultural context. A local CAB was convened and consisted of caregivers, community members, early childhood development professionals, school staff, and health professionals with various levels of Diné cultural knowledge to provide input to the +Language is Medicine curriculum modules. A Diné linguist/elder was a key CAB member who also provided translation work of +LiM intervention materials. 95% of the 23 CAB members identified as Diné. CAB members were partly identified through established community partnerships from previous and current JHCIH programs. Additional stakeholders, who were not early childhood professionals, teachers, or healthcare staff, were consulted to provide their parent and community insight. Early childhood professionals included individuals from early intervention programs (IDEA Part C) and other local THV programs servicing Diné families. CAB members were compensated for their time and participation in CAB meetings.

#### 2.1.5 Consult with stakeholders

The research team virtually met with CAB members throughout the development of the +LiM curriculum. During CAB meetings, the Principal Investigator reviewed +LiM intervention content, including lessons and visuals, and CAB members provided live feedback. There were three CAB meetings over 4 months, giving time for the research team to develop the curriculum and incorporate feedback. The lead PI and tribal home visiting curriculum experts on the research team utilized the two EBIs previously described (Hanen “It Takes Two to Talk” and EMT) for concept development of five lessons promoting language nutrition. In the first CAB meeting, members provided feedback on the following proposed lesson topics: (1) Back and Forth with your Child (Reciprocal and Responsive Communication), (2) Reading Together (Shared Book Reading), (3) Following Your Child’s Lead, (4) Encouraging Communication through Pretend Play, and (5) Incorporating Native Language into Daily Life. Open-ended questions to the CAB were utilized to facilitate discussion about the lesson concepts. Additionally, questions were asked to the CAB on engagement with young Diné families and how to best support Diné language use with infants and toddlers. In the second meeting, the team discussed a draft of the first lesson and asked questions to the CAB about the content, relatability, and flow of the lesson. Before the last meeting, lesson drafts were mailed to CAB members for review. In the last meeting, the +LiM team presented the remaining lessons for CAB feedback.

#### 2.1.6 Decide what needs enhancement

Native language use and cultural relevance throughout the +LiM curriculum were key areas for enhancing the two previously mentioned EBIs. This included initial +LiM team discussions on how to promote language nutrition within home and cultural routines. CAB members provided additional recommendations to the curriculum after reviewing the draft lessons.

#### 2.1.7 Enhance the original program

An enhancement plan was developed to incorporate CAB feedback into the full lesson drafts and training plan through the following steps: (1) compile, (2) apply, (3) design, (4) print, (5) train. CAB meeting feedback was compiled to highlight key edits and additions to lesson content and guided activities. The research team worked with the Family Spirit curriculum writer to apply the appropriate sections of each lesson with the cultural recommendations and additions. The evidence-based Family Spirit home visiting model was chosen due to its effectiveness in previous programs (11, 13, 24), and familiarity among the research team’s community-based home visitors. In the design step, graphic design elements were finalized to improve the consistency in look and feel of the lessons. Complete draft lessons were reviewed and discussed in team meetings for a final round of curriculum revisions before being printed for training use. The community-based +LiM home visitors provided additional recommendations for curriculum revisions during the training period. After the training period, final curriculum versions were printed for pilot study use.

#### 2.1.8 Train staff

The research team had prior experience in home visiting research practice and implementation. The community-based tribal home visitors were already trained and familiar with the delivery of the Family Spirit curriculum model. A precision THV approach was utilized to train field staff in the +LiM curriculum content. The PI trained the home visitors in administration of standardized child developmental assessments and curriculum intervention delivery. A combination of virtual and in-person meetings were utilized to train the tribal home visitors in data collection procedures and lesson delivery.

Pre-training in child developmental assessments was essential for understanding and recognizing developmental delays. Pre-training included review of developmental assessment materials which included the Ages & Stages Questionnaire – 3rd Edition [ASQ-3; (43)], and the Bayley Scales of Infant and Toddler Development – 4th edition (44), and a review of the procedure manuals. After an initial review of assessment materials, the PI utilized didactic teaching on administering and scoring these assessments. The home visitors shadowed virtual early intervention visits with the PI, followed by discussion and identification of language facilitation strategies from “It Takes Two to Talk” and Hanen. Following this observation of PI demonstration, a reliability training plan was developed for home visitors to practice administering assessments with Diné families. The PI and home visitors completed home visits together and completed assessment questionnaires individually, without coaching from the PI. Assessments were then scored and measured by the PI for reliability between the home visitors and the PI/SLP. The PI provided coaching and feedback after these practice home visits. The PI observed (live or video) home visitors in practice visits until they achieved 85% inter-rater reliability.

Final versions of the +LiM curriculum lessons were in development during the assessment training period prior to study launch. A series of training meetings were conducted with the home visitors on teaching the +LiM module. These meetings consisted of didactic teaching on key strategies of the +LiM intervention such as active listening, tailoring, Teach-Model-Coach, comprehension checks, and early intervention referrals. Home visitors also completed virtual role play sessions with the PI and team curriculum specialists which included live and retrospective coaching.

#### 2.1.9 Test the enhanced materials

The enhanced +LiM curriculum is being implemented through a pilot study. Lesson fidelity checks are being completed by the PI for home visitor content competency. Sharing of pilot study results will be determined after pilot study completion and evaluation.

### 2.2 Timeline

This process of enhancement took place from December 2021 through December 2022. This included convening and meeting with the CAB, drafting lessons and incorporating CAB feedback, and training tribal home visitors in administration of child developmental assessments and lesson delivery.

## 3 Results

As a result of this collaboration with key community members, our research team produced two main products that drive the +Language is Medicine pilot study: (1) a culturally responsive interpretation of language nutrition that centered Diné knowledge and beliefs and (2) a five-lesson language nutrition curriculum based on EMT principles, shared book reading, increasing pretend play, and incorporating native language into family routines.

### 3.1 Indigenous language nutrition

Language nutrition, which describes early language exposure rich in quality and quantity and delivered in home routines, naturally became a center point of topic during meetings with CAB members. Language use, in the context of caregiver-child interactions, language use in ceremony, and exposure to Native languages were common references during conversations with CAB members and the study team. Language use that centers cultural routines and language revitalization directly correlates with the nourishment and sustenance of a community’s linguistic heritage, cultural practices, and traditional ways of knowing (e.g., childrearing practices). In this context, language use was presented as a vital element of cultural identity, connecting individuals to their culture. The following themes were extracted from community-based conversations, in which the study team developed an approach for the enhancement of the two language facilitation strategies selected for the basis of the +LiM intervention (30, 31):

- Identifying naturalistic language-rich routines, such as using kinship terms, greetings, storytelling, and participating in ceremonies (e.g., Diné first laugh celebration)
- Aligning parent coaching topics with seasonal celebrations (e.g., planting and harvesting, winter games, birthday parties, and wood hauling)
- Alignment with Diné language revitalization efforts in early childhood programs based on local needs and family preferences
- Building on intergenerational learning and facilitating the transfer of language skills from elders to younger generations to foster linguistic knowledge and cultural understanding
- Embedding opportunities for discussion of traditional practices such as hunting, gathering, or craftsmanship which provides a context for language use that aligns with cultural values and caregiver practices
- Honoring Diné storytelling and oral traditions to preserve cultural narratives.

The process of establishing strong Indigenous language nutrition refers to nurturing the linguistic and cultural ecosystem in a holistic manner while acknowledging the interconnectedness of language, identity, and community well-being. Most critical, it is about sustaining and revitalizing a community’s Indigenous language in a way that resonates with the cultural practices and values, fosters a sense of self-determination and intergenerational healing, and empowers caregivers to speak to their children in nurturing and respectful ways. This interpretation of language nutrition was developed internally by the study team because of engaging with our CAB members, spending time in the community, and applying methods of decolonization to the EBI enhancement process and establishing a basis for true community-leveraged enhancement strategies. It is with this redefinition that the study team moved into product development, which included a culturally tailored language nutrition curriculum that incorporated EMT and the Hanen Approach through parent coaching lessons.

### 3.2 The +language is medicine curriculum

A key component of the +LiM curriculum is an enhanced teach, model, coach approach to emphasize cultural routines and native language exposure in supporting multiple dimensions of child development. Key elements of +LiM’s teach, model, coach are (1) use the Family Spirit home visiting model as a platform (2) engage caregivers as a child’s first teacher (3) teach basic language nutrition concepts and language facilitation skills (4) demonstrate skills (live or video) (5) coach caregivers in practicing skills (6) exploring families’ home, cultural routines and environment to identify opportunities to add concepts of Indigenous language nutrition, as previously described.

The +LiM curriculum consists of 5 lessons categorized into three content areas: introduction to +LiM and foundational knowledge (Lesson 1: Encouraging Conversations with Young Children), building strategies to support language acquisition (Lesson 2: Following Your Child’s Lead), and identifying opportunities to apply skills and strategies in daily life (Lesson 3: Reading Together; Lesson 4: Pretend Play and Communication; Lesson 5: Native Language and Your Daily Routine).

A routines mapping activity was embedded in each lesson for families to identify home and cultural routines through which language facilitation strategies could be practiced and expanded upon. The activity utilizes a routines circle map consisting of four quadrants to represent household, outdoors, cultural, and community spaces. The goal of the routines mapping activity is for caregivers to identify in their daily routines when and where they can reinforce Indigenous language nutrition and practice language facilitation strategies with their child. This activity takes place at the beginning of the lesson and is referenced throughout the home visit. The routines map is revisited again at the conclusion of the lesson The CAB provided additional examples of daily routines such as narrating to a child the process of making dough for frybread (household routine), using rocks, sticks, or mud to pretend play (outdoors), singing a song or reciting a prayer in Diné Bizaad (Navajo language) with child (other cultural spaces), and greeting relatives in Diné Bizaad (community). These familiar routines are optimal places and moments for promoting language learning and practicing language facilitation strategies such as narration, imitation, labeled praise, and modeling through self-talk, parallel talk, and focused repeating.

## 4 Implementation supports

We also developed an implementation guide for use during the training of home visitors and serves as a reference point for how to further enhance the +LiM lessons. To help support the standardization of +LiM lesson delivery, a treatment fidelity checklist was developed by the PIdial to use by peers who will observe home visits.

## 5 Discussion

Tribal Home Visiting (THV), such as the Family Spirit home visiting curriculum, has shown strong promise in improving child and parental outcomes in tribal communities. Dedicated federal funding allows for the sustainability of these programs as long-term solutions to improving child developmental outcomes. Most importantly, the dedicated programming for AI/AN families allow for cultural enhancement and further tailoring of caregiver coaching interventions. The +LiM team aims to deliver their +LiM curriculum within this existing infrastructure and to further develop developmental surveillance and response efforts. Due to the shortage of special education personnel in reservation communities, a precision home visiting approach can be utilized to help meet the need. In this case, the +LiM team seeks to have a wider reach of skilled SLP services delivered at the tertiary prevention level (e.g., Birth-to-Three programs) and aim to deliver primarily through secondary prevention efforts (e.g., THV). With this model, the SLP can have a wider reach to families in underserved areas of tribal nations. In addition to this precision public health model, incorporating EBIs into home and cultural routines can take place. With this wider reach and ability to tailor interventions, the team can utilize community engagement to incorporate community values, practices, and traditions. This enhancement process demonstrates how EBIs used with young children with DD can be modified and adapted to incorporate cultural values and support native language use. Most importantly, this paper highlights the critical steps necessary to ensure that tribal community input is the driving factor of EBI cultural enhancement. Assessment of preliminary outcomes of enhancing EMT and Hanen for use with Diné toddlers with DD is the next major step for the +LiM research team and will be shared through future publications and community-based presentations.

## Data availability statement

The original contributions presented in the study are included in the article/supplementary material, further inquiries can be directed to the corresponding authors.

## Author contributions

TB: Writing – original draft, Writing – review & editing. EK: Writing – original draft, Writing – review & editing. JM: Writing – original draft, Writing – review & editing. LM: Writing – original draft, Writing – review & editing. LJ: Writing – original draft, Writing – review & editing. MA-G: Conceptualization, Methodology, Writing – review & editing. AB: Writing – original draft, Writing – review & editing. JA-B: Conceptualization, Data curation, Formal analysis, Funding acquisition, Investigation, Methodology, Project administration, Resources, Software, Supervision, Validation, Visualization, Writing – original draft, Writing – review & editing.
